# Cancer of the Breast and Its Operative Treatment

**Published:** 1907-09

**Authors:** 


**Cancer of the Breast and its Operative Treatment.** By W. SAMPSON HANDLEY. Pp. xii., 232. London: John Murray. 1906.

The aim of the author is "to present for the surgeon's use a careful picture of a breast cancer, of its microscopic ramifications, and of its mode of dissemination, and upon this basis to discuss the methods of operative treatment." Questions of ætiology, pathogenesis, and diagnosis are not considered.

The greater part of the work is devoted to the development of an original hypothesis, that of "lymphatic permeation," to explain the mode of dissemination of cancer of the breast.

By this hypothesis, which is likely to be handed down to posterity as "Handley's (Lymphatic) Permeation Hypothesis," the master process in the dissemination of cancer of the breast is the spread of the growth along the fine lymphatic vessels of the parietes, independently of the transport of cancerous particles by the lymph and blood streams. The existence of the latter mode of dissemination, the "embolic" mode, is admitted, but it is not considered to be the chief process at work. The chief process is a tendril-like growth of cancer from the primary deposit in all directions in the fascial plane along the lymphatics, the growth being independent of the current of lymph. In the parietes the extension is not primarily in the skin, but in the "fascial lymphatic plexus," which lies in the subcutaneous fat upon the deep fascia.

After giving a lucid and excellently illustrated account of the macroscopic and microscopic investigations upon which the permeation hypothesis has been founded, the author proceeds, in the final chapters, to enunciate the principles which should underlie the operative treatment of breast cancer, and he suggests modifications of technique which are likely to improve the results of these operations if his hypothesis of dissemination represents the truth.

In our opinion it is the plain duty of every surgeon who operates for mammary cancer to give due consideration to the views which the author has advanced. We believe that in time all surgeons will become acquainted with them more or less thoroughly, for they seem to be endowed with "permeative" force. We advise that the acquaintance be made a thorough one at once, through the medium of the author's book itself. If we mistake not, the investigations he has recorded will mark an important epoch in the history of the campaign against cancer of the breast; but whether that be so or not, the fact remains that the book is one of conspicuous merit and fascinating interest.

